# The Effect of Type of Delivery on Female Postpartum Sexual Functioning: A Systematic Review

**DOI:** 10.3390/healthcare10071212

**Published:** 2022-06-28

**Authors:** Effrosyni Nikolaidou, Evangelia Antoniou, Angeliki Sarella, Maria Iliadou, Eirini Orovou, Maria Dagla

**Affiliations:** Department of Midwifery, School of Health & Care Sciences, University of West Attica, 12243 Athens, Greece; nikolaidou.ef@gmail.com (E.N.); lilanton@uniwa.gr (E.A.); asare@uniwa.gr (A.S.); miliad@uniwa.gr (M.I.); eorovou@uniwa.gr (E.O.)

**Keywords:** sexual function, type of delivery, vaginal delivery, C-section delivery, FSFI

## Abstract

Female sexual function could be considered as multifactorial. Specific physiological structures and hormonal fluctuations postpartum, along with the psychological adjustment of women, could result in altered sexual function. The primary aim of this review was to systematically appraise the existing data on the effect of mode of delivery on female sexual function. This review was designed based on the PRISMA statement guidelines. An extensive literature search was performed in the Pubmed, Scopus, and PsycInfo databases, using prespecified inclusion/exclusion criteria, between the 20 September and 10 October 2021. Studies’ quality assessment was conducted using the Quality Assessment Tool for Observational Cohort and Cross-Sectional Studies of the National Heart, Lung, and Blood Institute. The initial search involved 1592 studies. The last step of the screening procedure yielded 16 studies, including 41,441 subjects with a mean age of 29.9 years. Studies included groups with spontaneous and assisted vaginal and C-section delivery modes. No statistically significant difference between groups was found. The type of delivery appears to be irrelevant regarding this relationship. Moderating factors seemed to indirectly influence this relationship. Health professionals should educate expectant mothers and be aware of the possibility that delivery method could affect sexual function.

## 1. Introduction

According to the World Health Organization, sexual health refers to a respectful approach to sexual expression and the ability to experience pleasure, while it simultaneously constitutes one of the essential dimensions of well-being which affects the health of individuals globally [1]. Data on the incidence of female sexual dysfunction could be characterized as limited, given that different studies report different rates ranging from 20% to 70% in the general population, with the majority of participants facing difficulties with desire and arousal [2].

Female sexual function could be considered as multifactorial, affected by biological, psychological, interpersonal, and social factors [3]. Among the biological factors, specific physiological structures and hormonal fluctuations are considered as key elements of sexual function. During sexual arousal and intercourse, increased blood flow causes genital vasocongestion. Distension of the vaginal wall provokes pressure in the interiors of capillaries, which, through the vaginal epithelium, results in plasma perspiration [4]. Furthermore, the role of estrogen levels in the acidic environment, as well as in the general regulation of female sexual function, is well-documented [5]. Psychological factors affecting sexual function include the individual’s dominant personality traits, and overall mental health state [6], whereas interpersonal factors include the thesis of the individual in romantic relationships, past sexual experiences, and the way affection and physical intimacy were learned to be expressed [7]. With respect to social factors, these include expectations that the individual has developed through religious, societal, and cultural norms [7]. 

During childbirth, remarkable changes occur with respect to vaginal structure [8]. Research has shown that one in five women giving birth suffer from dyspareunia during the first three postpartum months [9], while another study reported that physically-related sexual problems were present in over 80% of women after childbirth [10]. Findings have indicated that perineal trauma and, consequently, the delivery method were associated with sexual problems in 80% of women [11]. Likewise, it has been shown that one of the factors affecting the resumption of sexually intimate relationships for postpartum women is the level of perineal injury occurring during delivery [12]. Hence, reporting on the effect of delivery method on female sexual function could assist clinicians in informing women regarding the impact of delivery type on their sexual function. 

The prevalence of sexual dysfunction has been explored and highlighted in several studies [13], since a healthy sexual life can be characterized as a cornerstone of an individual’s overall health [14]. Simultaneously, it has been shown that, after childbirth, specific changes occur depending on the type of delivery. With respect to vaginal delivery, whether this is spontaneous or assisted delivery, perineum trauma and pelvic floor alterations are among the consequences, while chronic postpartum pain appears to affect 6–11.5% of women [15], which could negatively affect postpartum sexual function. Meanwhile, cesarean delivery has fewer implications regarding sexual life, and has been associated with better sexual performance [16].

To the authors’ knowledge, up to the date this article was written, no systematic review on the effect of type of delivery on female sexual function was published within the last decade. The primary aim of the present study was to systematically appraise the existing knowledge and evaluate the association between the type of delivery and female sexual function postpartum. 

## 2. Materials and Methods

The present systematic review aims to compile all available data on the association between the type of delivery and female sexual function. The review’s design was based upon the Preferred Reporting Items for Systematic Reviews and Meta-Analyses (PRISMA) statement guidelines [17] to identify those papers with a relevant topic. Basic stages of the review involved the formulation of the research question and an extensive search of the existing literature in the field. Following that, data extraction and evaluation were conducted, and lastly, data analysis and presentation of information were documented. Studies included in this review followed specific eligibility criteria as indicated below. 

### 2.1. Eligibility Criteria

For a study to be eligible, it had to comply with specific inclusion/exclusion criteria. The study had to evaluate solely the female postpartum sexual function. Adult-only participants, regardless of sexual orientation and relationship/marital status, were required to participate in this study. The study groups had to derive from the general population and not focus on subjects with sexual dysfunctions established prior to the childbirth. The postpartum period, according to the American College of Obstetricians and Gynecologists, may last up to 12 months post-delivery [18]. The assessment of sexual function had to be evaluated within this timeframe. Additional measurements at a later time did not constitute a reason for exclusion. Given that cesarean delivery in some cases is emergent, complications during pregnancy and childbirth were not reasons for exclusion. Sexual function had to be evaluated using established tools in studies (e.g., the Female Sexual Function Index). Studies using inquiries structured by research teams were excluded. For studies to be eligible for inclusion, they had to be published in the English language from peer-reviewed journals within the last decade. Regarding the exclusion criteria, given the fact that the World Health Organization sets the reproductive age at 15–49 years, papers including women over the age of 49 were excluded. Studies that included subjects with mental illnesses were excluded because of the effect that systematic psychotropic medication can have on sexual function [19]. Other systematic reviews or meta-analyses were not included, and research protocols that did not provide sufficient data were also excluded.

### 2.2. Search Strategy

Pubmed, Scopus, and PsycInfo databases were thoroughly searched for relevant studies from 20th September to 10th October 2021. Research was conducted by two reviewing investigators using the following terms: “female sexual function” OR “female sexual dysfunction” OR “sexual activity” OR “female sexual health” AND “type of delivery” OR “mode of delivery” OR “vaginal delivery” OR “cesarean delivery” and were adopted accordingly when necessary. As an example of the search procedure, the syntax used in the Pubmed database was ((female sexual function) OR (female sexual dysfunction) OR (female sexual activity)) AND ((mode of delivery) OR (type of delivery) OR (vaginal delivery) OR (cesarean delivery)). Titles, keywords, and abstracts of each study were screened for eligibility. A backward search (hand search of reference lists) of included papers was conducted to identify additional studies relevant to the topic. All studies found were assessed according to the eligibility criteria. 

### 2.3. Data Extraction and Quality Evaluation

To evaluate each paper, specific data were extracted from each included study. These data included the study’s first author and the year of publication, the sample size of each study divided in subgroups based on type of delivery, participants’ mean age, any measurements applied to evaluate the factor of sexual function, the main outcomes of individual studies and any information required for the quality evaluation. The Quality Assessment Tool for Observational Cohort and Cross-Sectional Studies of the National Heart, Lung, and Blood Institute was used to assess studies’ quality [20]. The tool consists of fourteen items evaluating observational (cohort and cross sectional) studies. The aim of the tool is to assist the systematic interpretation of an observational study. Each question of the tool can be answered with a Yes, No, or Other (CA, cannot determine; NA, not applicable; NR, not reported), investigating whether the criterium under investigation is fulfilled. However, it must be noted that this tool is not used to generate a total quality score due to the well-known problems associated with such scores [21]. Nevertheless, it aims to examine a study’s overall quality and consider the risk of bias. The procedure of data extraction was performed by two reviewers.

## 3. Results

**Figure 1:**
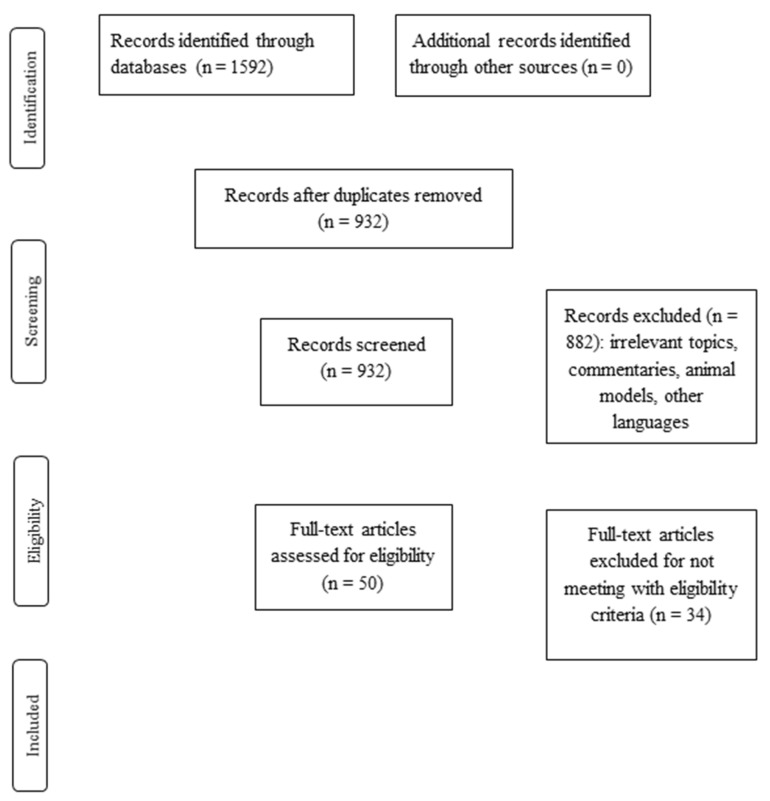
Flow diagram of the included studies.

The initial search of the literature found 1592 studies. After removing all papers on irrelevant topics, and applying the inclusion and exclusion criteria, the final step of the screening procedure yielded 16 studies. The complete screening process is presented in Figure 1.

### 3.1. Basic Characteristics of the Included Studies

All of the studies were observational, with a cohort or cross-sectional design. In total, the included studies recruited 41,441 subjects with the largest sample being n = 37,417 and the smallest being n = 49. The mean age of all participants was 29.9 years old for 11 of the included studies, as five of them provided solely the age range (18–45) for their subjects. All 16 studies used self-report instruments to measure sexual function; 13 of them used the Female Sexual Function Index (FSFI), 2 of them used other validated and well-established instruments to measure sexual function (Index of Sexual Satisfaction, Sexual Function Questionnaire’s Medical Impact Scale, Female Sexual Function Questionnaire), and 1 study, apart from a self-report measure distribution, applied an experiment to assess genital response using Laser Doppler imaging. The basic characteristics of the included studies are presented in Table 1. 

### 3.2. Main Results Based on the Research Question

Among the included studies, 10 compared cesarean delivery with vaginal delivery, clarifying whether vaginal delivery was spontaneous or operative. Four of them performed three-group comparisons between cesarean, operative vaginal, and spontaneous vaginal delivery, one of them performed between-group comparisons of cesarean delivery, vaginal delivery and nulliparous women and one compared spontaneous vaginal delivery with operative vaginal delivery. 

With respect to the effect of delivery type on the sexual function, there was no statistically significant difference between cesarean and vaginal delivery in 10 studies [12,22,23,24,25,26,27,28,29,30,31,32,33]. Among the remaining six studies, one found no statistically significant difference between groups apart from the arousal subscale [28]. One of the studies reported significantly lower scores, but solely for a specific subscale of the instrument used (existence of partner or not) [34]. One of them found a significant difference in favor of the vaginal delivery group [35], while one of them found the exact opposite (in favor of the cesarean delivery) [36]. For the one study comparing operative and spontaneous vaginal delivery, results were statistically different only for one of the measurements (at 3 months postpartum) in favor of the spontaneous vaginal delivery group, while no difference was found at 6- and 12-month postpartum assessments [25]. One last study found significantly lower scores only in the satisfaction and pain subscales. However, this applied only to the women who gave a vaginal birth and received an episiotomy [31]. 

A number of studies performed repetitive measurements of sexual function at later time points in order to evaluate after which type of delivery sexual function was restored faster [25,31,32]. In addition, other studies tried to investigate the role of mediating factors and it appeared that the mode of delivery negatively affected postpartum sexual function when the childbirth experience was perceived as negative [29] and when the degree of perineal trauma was more severe [25]. 

### 3.3. Quality Evaluation

Quality evaluation was conducted by two reviewers with the use of the Quality Assessment Tool for Observational Cohort and Cross-Sectional Studies. Overall quality did not significantly vary across studies, with most of them being of moderate to high quality. The main issue was the lack of sample justification, as most studies recruited convenience samples. The risk of bias regarding internal consistency could be characterized as low since all the included studies used validated instruments to evaluate the outcome of interest. Detailed outcomes of the quality evaluation are presented in Table 2.

## 4. Discussion

The aim of this study was to systematically present all available data on the effect that the type of delivery has on female sexual function postpartum. To the authors’ knowledge, no similar article has been published within the last decade without strict criteria, such as the specificity of origin for the included studies. 

Based on the whole body of data that was gathered for this review, it appears that the type of delivery does not affect the postpartum sexual function. Overall, no significant difference was found in either global sexual health or aspects of it, apart from minor exceptions. The same applied even when the studies repeatedly measured sexual function at different time points of 6, 12, or even 24 months postpartum. Though the results show that there is no significant relation between sexual function and type of delivery, it appears that specific situations act as moderating factors in this relationship. One study revealed the significant indirect effect of the experience of childbirth on sexual function highlighting the importance of psychological wellbeing. Those who received an emergency C-section delivery or an operative vaginal delivery reported a worse childbirth experience, and those with a worse childbirth experience reported lower sexual function. This is consistent with the existing literature highlighting the effect of delivery on mothers’ birth experience [37], and simultaneously, it underlines the possibility of the experience being affected, not by the mode of delivery itself, but by the occurring emergency. Another mediating factor appeared to be the extent of the perineal trauma. Research has shown that severe perineal trauma is present for about 3% of women within European countries and up to 19% in the United States of America [38]. Perineal trauma is associated with the rise of dyspareunia, one of the classified female sexual disorders [39]. It has been shown that severe perineal trauma is related to a longer time needed for sexual activity resumption and deteriorated sexual function [40]. Another systematic review explored additional components as possible risk factors of female sexual dysfunction after childbirth. The authors of that review outlined that the type of delivery does not affect sexual function. However, factors such as the degree of perineal trauma and whether the mother breastfed the child appeared as influencing characteristics [40].

A healthy sexual life can be characterized as a cornerstone of an individual’s overall health [14], and a number of researchers have tried to clarify whether the type of childbirth can affect it. As a matter of fact, there are somewhat similar findings to this study. For example, a recent meta-analysis on the sexual function of postpartum women in China found differences in the compared groups with respect to sexual activity resumption and the pain experienced during intercourse in favor of cesarean delivery, but this difference became insignificant as time passed between 3 and 6 months postpartum [41]. Likewise, another systematic review on this topic, conducted ten years ago, resulted in inconclusive findings. Its results report no significant difference in the sexual function of women with vaginal and cesarean delivery. Similar to this report, it appeared that independent factors, such as performing an episiotomy with or without additional trauma of the perineum, had an increased possibility of presenting the sexual disorder of dyspareunia [42]. 

This study includes some strong points such as strict eligibility criteria, which ensure that misleading factors, such as sexual disorder diagnoses established prior to the pregnancy or delivery, are excluded. Nevertheless, it simultaneously bears certain limitations. In most studies, no sample size calculation was performed in advance. Therefore, their generalization could only be performed with caution. Furthermore, the body of evidence (excluding one study) derived from measurements with self-report instruments. This makes the findings vulnerable to reporting bias. In addition, the majority of the included studies were of cross-sectional design, which means that the cause–effect relationship might have been biased by unpredicted factors. These limitations render the generalization of the findings impossible to some degree.

## 5. Conclusions

This review attempted to systematically approach and present all available data from the last decade with respect to the effect of the type of delivery on female sexual function. The type of delivery appears irrelevant regarding this relationship. Moderating factors, such as the subjective experience of childbirth or the extent of perineal trauma, seem to indirectly influence this relationship. However, health professionals should be aware of the potential the delivery has to affect sexual function, and they should educate expectant mothers. Future studies with sample size calculation beforehand, even of cohort design, could better depict the relationship between the type of delivery and sexual function postpartum, excluding any possibly misleading factors. 

## Figures and Tables

**Table 1:** Basic characteristics of the included studies of this systematic review.

Author, Year, Country of Origin	Sample Size (n)	Mean Age ± SD *	Type of Delivery Comparison (n of Group)	Measurements of Outcome of Interest	Main Results
**Handelzalts, 2018, Israel**	376	30.8 ± 4.2	CD (elective and emergency) (92), VD (spontaneous and instrumental) (284)	CPQ, ISS, SFQ-MIS	No direct effect of delivery mode on sexual function was found. Indirect effect of delivery mode was found for sexual functioning experience [B = −0.26, *p* = 0.023, 95% CI = (−0.40, −0.10)] and sexual satisfaction [B = 0.11, *p* = 0.013, 95% CI = (0.05, 0.21)] both mediated by childbirth experience.
**Amiri, 2017, Iran**	203	24.91 ± 4.9	CD (113), VD (90)	FSFI	No statistically significant difference was discovered between the groups of comparison.
**Ghorat, 2017, Iran**	177	31.81 ± 6.31	CD (54), SVD (123)	FSFI	No statistically significant difference was discovered between the groups of comparison (*p* = 0.23).
**Lurie, 2013, Israel**	82	18–45	Vaginal without episiotomy (16), vaginal with an episiotomy (14), instrumental delivery (16), emergent C-section (19), elective C-section (17)	FSFI	Total scores of FSFI did not show significant difference at 6, 12 or 24 weeks postpartum.
**De Souza, 2015, Australia**	131	30.70	CD (28), SVD (77), OVD (26)	FSFI	In total or subscale scores of FSFI, no significant difference was found between groups at antenatal and 12 months postpartum assessments.
**Banaei, 2018, Iran**	361	28.21 ± 4.22	CD (181), VD (180)	FSFI	No significant difference was found between groups of comparison (*p* = 0.07).
**Cappell, 2020, Canada**	49	31.45 ± 4.35	CD (15), VD (16), Nulliparous (18)	Laser Doppler imaging was used to assess genital response while participants watched a neutral and erotic film, FSFI	Genital response and subjective sexual arousal/response was not significantly related to mode of delivery. VD group had significantly lower flux units compared to CD group (*p* = 0.05).
**Song, 2014, Japan**	435	33.2 ± 4.4	SVD (282), OVD (21), planned CD (23), emergency CD (27)	SFQ28	Only the partner subscale of the instrument revealed significant difference between groups.
**Hjorth, 2019, Denmark**	37,417	18–45	SVD (25,646), OVD (5460), CD (6311)	Structured inquiry on sexual health, adapted from the Danish National Health Survey	Significantly fewer sexual problems only for the VD group following a C-section delivery.
**Saleh, 2019, Egypt**	684	29.0 ± 6.2	CD (364), VD (320)	FSFI	Women with a history of CS had statistically significant higher FSFI total score compared to VD (31.3 ± 3.8 vs. 30.23 ± 3.6, respectively; *p* < 0.001).
**Barbara, 2016, Italy**	269	32.4 ± 4.9	SVD (132), CD (92), OVD (45)	FSFI	OVD showed significantly lower FSFI total scores and subscales (arousal, lubrication, orgasm) compared to CD, and stat. sign. lower score in orgasm with SVD. The mode of delivery did not affect the resumption of sexual intercourse.
**Kahramanoglu, 2017, Turkey**	403	18–45	CD (138), VD and episiotomy (265)	FSFI	Significant within groups differences in specific subscales of FSFI at 3-month assessment. No significant difference between groups at 6-, 12-, or 24-month assessments.
**Dabiri, 2014, Iran**	150	27.87 + 5.64	CD (69), VD (81)	FSFI	No significant differences between mode of delivery and sexual functioning in total scores or subscales
**Eid, 2015, Egypt**	200	29.1 ± 3.11	CD (110), VD (90)	FSFI	The mode of delivery had insignificant effect on the FSFI at 12 weeks postpartum.
**Saydam, 2019, Egypt**	142	18–45	CD (77), VD (65)	FSFI	No difference was found between CD and VD groups.
**de Sousa, 2021, Portugal**	196	26–36	OVD(131), SVD(65)	FSFI	Significantly lower FSFI total score for the OVD group (21.3 ± 8.6 vs. 24.9 ± 7.9, *p* = 0.015) at 3 months. At 6 months, there were no differences in FSFI scores based on the type of delivery. At 12 months, similar total FSFI score between groups, except for the pain subscale (*p* = 0.004) in favor of the SVD group. Perineal trauma was independently associated with sexual dysfunction (*p* = 0.02) at 3 months.

* Age is presented in mean  ±  standard deviation or minimum–maximum. ABBREVIATIONS: CD = cesarean delivery; VD = vaginal delivery (when not clarified if spontaneous or operative); OVD = operative vaginal delivery; SVD = spontaneous vaginal delivery; CPQ = Childbirth Perception Questionnaire; ISS = Index of Sexual Satisfaction; SFQ-MIS = Sexual Function Questionnaire’s Medical Impact Scale; FSFI = Female Sexual Function Index; SFQ28 = Female Sexual Function Questionnaire.

**Table 2:** Quality assessment of the included studies based on the Quality Assessment Tool for Observational Cohort and Cross-Sectional Studies.

	Study															
Item	Handelzalts, 2018	Amiri, 2017	Ghorat, 2017	Lurie, 2013	De Souza, 2015	Banaei, 2018	Cappell, 2020	Song, 2014	Hjorth, 2019	Saleh, 2019	Barbara, 2016	Kahramanoglu, 2017	Dabiri	Eid, 2015	Saydam, 2019	De Sousa, 2021
1. Clear research question	Y	Y	Y	Y	Y	Y	Y	Y	Y	Y	Y	Y	Y	Y	Y	Y
2. Population clearly defined	Y	Y	Y	Y	Y	Y	Y	Y	Y	Y	Y	Y	Y	Y	Y	Y
3. Participation rate > 50%	Y	Y	Y	Y	Y	Y	Y	Y	Y	Y	Y	Y	Y	Y	Y	Y
4. Prespecified eligibility criteria	Y	Y	Y	Y	Y	Y	Y	Y	Y	Y	Y	Y	Y	Y	Y	Y
5. Sample size justification	N	Y	N	Y	Y	Y	N	N	N	N	N	N	N	N	N	N
6. Exposure of interest measured before the outcome	NA	NA	NA	NA	NA	NA	NA	NA	NA	NA	NA	NA	NA	NA	NA	NA
7. Sufficient timeframe between exposure and outcome	Y	Y	Y	Y	Y	Y	Y	Y	Y	Y	Y	Y	Y	Y	Y	Y
8. Different levels of exposure examination related to the outcome	Y	Y	Y	Y	Y	Y	Y	Y	Y	Y	Y	Y	Y	Y	Y	Y
9. Exposure measures clearly defined	Y	Y	Y	Y	Y	Y	Y	Y	Y	Y	Y	Y	Y	Y	Y	Y
10. Exposure assessed more than once over time	NA	NA	NA	NA	NA	NA	NA	NA	NA	NA	NA	NA	NA	NA	NA	NA
11. Outcome measures clearly defined	Y	Y	Y	Y	Y	Y	Y	Y	Y	Y	Y	Y	Y	Y	Y	Y
12. Outcome assessors blinded to the exposure	NA	NA	NA	NA	NA	NA	NA	NA	NA	NA	NA	NA	NA	NA	NA	NA
13. Loss to follow-up after baseline < 20%	Y	Y	Y	Y	Y	Y	Y	Y	Y	Y	Y	Y	Y	Y	Y	Y
14. Confounding variables measured and adjusted statistically	Y	N	Y	Y	Y	N	Y	Y	Y	N	Y	N	N	N	N	Y

**ABBREVIATIONS**: Y = yes; N = no; NA = not applicable.

## Data Availability

Not applicable.
